# On the π Coordination of Organometallic Fullerene Complexes

**DOI:** 10.3390/molecules16064652

**Published:** 2011-06-03

**Authors:** Bertha Molina, Liliana Pérez-Manriquez, Roberto Salcedo

**Affiliations:** 1 Facultad de Ciencias, Universidad Nacional Autonoma de México, Apdo. Post 70-646, Coyoacán 04510, Mexico; 2 Instituto de Investigaciones en Materiales, Universidad Nacional Autónoma de México, Circuito exterior s/n, ciudad universitaria, Coyoacán 04510, Mexico

**Keywords:** C_80_, organometallic complexes, theoretical calculations

## Abstract

Novel organometallic complexes of fullerene C_80_ and aryl ligands were simulated. The nature and characteristics of this family of complexes involving π coordination between the fullerene and a metal centre have been studied from a theoretical point of view. We are particularly interested in complexes where η^6^ coordination is present, this being the strangest manifestation of known coordinations, and thus we have studied several known and simulated compounds of this kind in order to understand the lack of examples. The presence of other η^6^ or η^5^ ligands on the opposite side seems to be an important element aiding the stabilization of these complexes, also inducing the conductive and semiconductive behaviour of the studied species.

## 1. Introduction

The extensive family of fullerene compounds has attracted considerable interest in several different fields of chemistry and physics due to their particular intrinsic characteristics [1,2]. One of the many themes related to this topic is the relationship between fullerenes and metal atoms, particularly in the case of exo coordination chemistry [3,4,5].

There are several examples of coordination compounds where a fullerene is joined to a metal atom, but the large majority are compounds where the coordination occurs in η^2^ fashion on a 6,6 junction of the fullerene [3,6], although there are several examples of fullerene-metal coordination compounds where the η^5^ mode is found [7] and an experimental result where there is an η^6^ complex [8,9].

All the references mentioned above concern experimental or theoretical works where the substituting fullerene is a C_60_ or a C_70_ unit, but there is also a very interesting possibility [3,10,11,12,13] concerning the design and preparation of new organometallic complexes where the ligand may consist of a larger fullerene. If this were the case, the advantage would be that larger fullerenes manifest a less pronounced degree of pyramidalization than that found in the case of the C_60_ or C_70_ mentioned above, therefore there is a greater possibility for η6 coordination to take place. The pyramidalization angle is defined as the angle between the projection of a specific C-C bond and a plane containing one of the designed carbons and its two adjacent atoms and it is useful to measure the sphericity of the fullerene molecules.

All the known metallic complexes consisting of larger fullerenes are endohedral, but there are no experimental reports describing an exo complex η^6^ with a C_74_ or larger ligand [3,4,5], but neither is there any study describing the capabilities of certain larger fullerenes for participating in the bonding with transition metal atoms in exo fashion; thus our aim was to undertake a study of this kind.

The main goal in this work is to examine the family of all C_80_ fullerene isomers [14,15,16] coordinated in η^6^ fashion with a transition metal atom, with an oxidation state of zero and complemented by a benzene ring, in order to have a coordination sphere which matches the 18 electron rule [17]. This study analyzes the electronic structure of the proposed complexes, as well as the possibility of stabilizing these species. A comparison of all the possible isomers is also undertaken.

## 2. Methods

All calculations were carried out by applying a pure DFT method for energy evaluations, applying Becke’s gradient corrections [18] for exchange and Perdew-Wang’s for correlation [19]. This is the scheme for the BPW91 method which forms part of the Gaussian03 [20] Package. All calculations were performed using the 6-31G** basis set. Frequency calculations were carried out at the same level of theory in order to confirm that the optimized structures were at a minimum of the potential surfaces.

## 3. Results and Discussion

**Figure 1:**
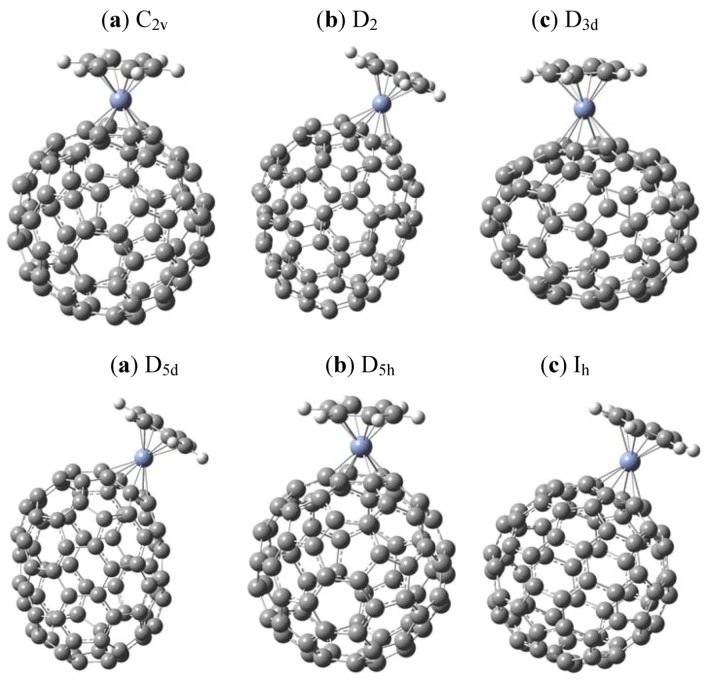
C_80_ isomers under study.

The C_80_ fullerene family consists of several members, and seven possible isomers have been identified [14]. They belong to the point groups D_5d_, D_2_, I_h_, C_2v_ (two isomers), D_3_ and D_5h_. We have simulated the possible coordination compounds of six of these and in the case of the fullerene C_2v_, we have found almost the same energy behaviour, as well as frontier molecular orbital behaviour. Therefore we have decided to include only the results from one of the two analogs. All the corresponding structures are presented in Figure 1.

It is obvious that cases exist where it is possible to find several isomers for the complexes because the metals have different coordination possibilities. To simulate all possibilities represents an enormous task and will be undertaken in a future study. However in this study, we present the results for only one structure from each fullerene isomer and we have chosen those instances where the structure was easier to optimize and where the six-member ring presents the most planar shape. We have adopted a nomenclature where each complex is named according to the point group of the fullerene that is coordinated with the metal, although in all cases the symmetry of the complexes is less than that of the isolated fullerenes.

The first important question is; can these complexes exist? If the answer is yes, can all isomers have complexes? In this respect, the entire family has been studied. The first analysis only consists of a comparison between the frequency calculations.

The results show that two species, *i.e.*, the isomers I_h_ and D_5h_ have negative frequencies, which was to a certain degree anticipated in both cases because these C_80_ isomers are not stable molecules [14,21] due to their large symmetry. This causes Jahn Teller distortions which promote changes that compel these configurations to change to one of the other isomers, mainly D_2_ and D_5d_. However it is very common to find that a compound becomes stable when it forms a complex with a metal centre, indeed both isomers can be stabilized by the formation of an endohedral complex with two atoms of a lanthanid metal [14,22], therefore we include both isomers in this study even though this may be difficult to prepare for experimental purposes.

The total energy of the six compounds can be compared; the relative values are presented (all referred to the isomer with the minimum energy, *i.e.*, the C_2v_). Thus the result is very interesting because again only the large symmetry isomers (I_h_ and D_5h_) seem to be difficult to prepare (in terms of thermodynamics). All present reasonable stability values, however there are other criteria which may be useful indicators.

The energy of the π bond between fullerenes and the chromium atom was calculated taking advantage of the theoretical results of all the species and considering the decomposition procedure described in Equation 1:

C_80_CrC_6_H_6_ → C_80_ + CrC_6_H_6_ (1)

The definitive results are presented in Table 1. The most noteworthy result is that one of the more stable isomers (D_5d_) manifests the strongest bond between the fullerene ligand and the metal.

**Table 1:** Relative data of the six isomers.

Isomer	π bond energy (kcal/mol)	E_rel _ (kcal/mol)	Angle (^o^)
D_3d_	25.54	2.95	20.9
I_h_	27.61	18.2	28.3
C_2v_	28.24	0.0	32.6
D_2_	33.15	1.75	33.4
D_5d_	37.01	2.38	33.0
D_5h_	29.30	23.22	28.9

The strength of this bond must be a consequence of improved overlapp between the orbitals of the metal and the six-member ring from the fullerene ligand, which participates in the coordination. A better interaction can be found for this isomer which has a larger pyramidalization angle, because the spheroidal shape of the body compels the pz orbitals of the six member rings on the fullerenes to become outside to the centre of the hexagone, this interaction can be found in the LUMO of all cases causing improved overlapping with the toroidal ring of the molecular orbitals dz^2^ emanating from the atomic centre of the Cr-benzene fragment localized in the HOMO of all configurations, considering the narrow difference between HOMO and LUMO in all cases the back bonding depicted is possible, however the effect is stronger in the case in which the pyramidalization angle is the larger.

**Figure 2:**
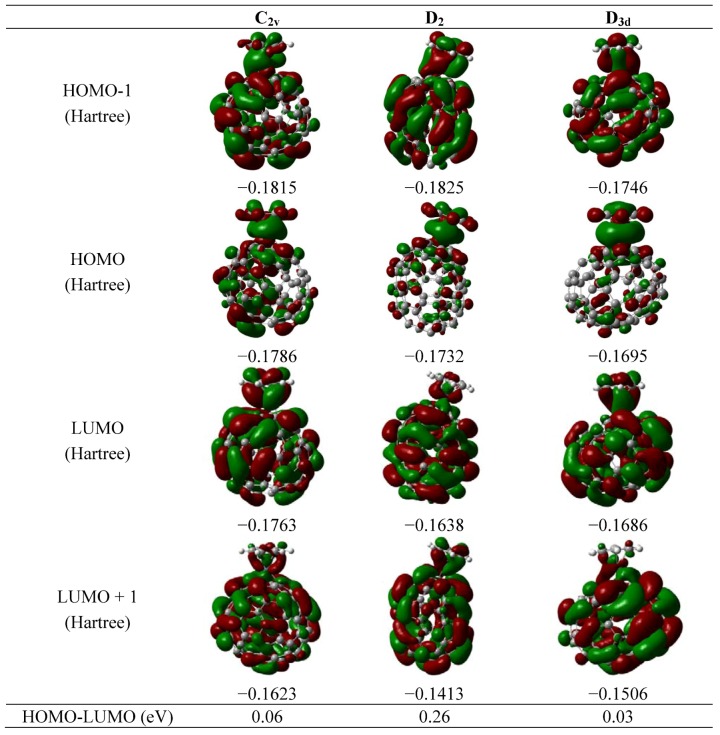
Frontier molecular orbital of the isomers C_2v_, D_2_ and D_3d_.

**Figure 3:**
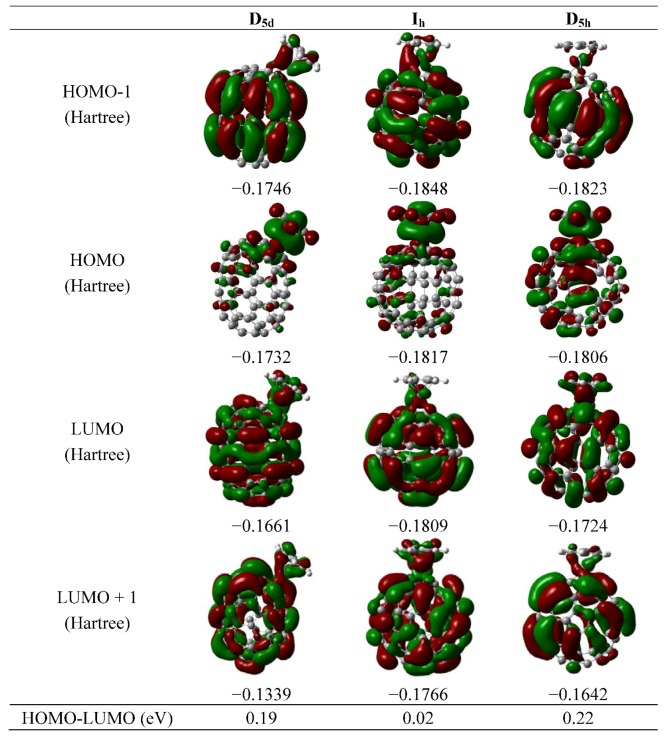
Frontier molecular orbital of the isomers D_5d_, I_h_ and D_5h_.

Therefore in the present cases a larger pyramidalization angle helps to have a better bonding between the fullerene and the metal atom. The shape of the resultant molecular orbitals for the complex is shown in Figure 2 and Figure 3. This feature [23] has been used as a criterion for evaluating the ability of fullerenes to coordinate in hapticities other than η^2^. In the case of the fullerenes from the C_80 _family, the corresponding values are presented in Table 1. The D_5d_ and D_2_ isomers have large pyramidalization angles. The same angle in the case of the fullerene C_60_ is 31°, a value which was obtained in both theoretical and experimental works [23,24].

The nature of the bond can be surmised as a consequence of the electronic behaviour of the fragments involved. First, all fullerenes and their derivatives are well known as electronic acceptors [25]. The symmetry and confinement of spheroidal molecules permit these compounds to have a very particular arrangement in terms of their molecular orbitals; indeed molecules belonging to the point group I_h_ are able to have a five folded degenerated set for the HOMO and a four folded degenerated set for the LUMO [26]. Thus in this case, there are many electrons in the frontier orbitals, the electronic gaps are likely to be short and the LUMO’s generally comprise very exposed MO’s.

The analysis of the molecular orbital scheme for all the isomers being studied is very important if a stable complex is to be proposed. All the frontier molecular orbitals are shown in Figure 2 and Figure 3. The original point groups from the spheroidal molecules alone is lost in the complexes, only two of the resultant molecules retains a small amount of symmetry, first the analogue I_h_ belongs to the point group C_2_ and the D_3d_ belongs to the C_s_ point group, therefore there are no cases with degenerated sets for their frontier orbitals, but in all cases the HOMO-LUMO gap is very short. These values are also presented in Figure 2 and Figure 3. These values are those of a conductor species, however they should be handled with care because it is possible that many of the isomers may be so unstable so that their structures attempt to change in order to become more stable and it would appear that this species may consist of the D_5d_ structure, where the value of the HOMO-LUMO gap for these species is ~0.2 eV, representing a conductor with a similar value to that of the grapheme [27]. This result is of great importance because the preparation of this compound will produce a compound with very interesting electronic properties.

## 4. Conclusions

The ability of the family of C80 fullerenes to generate organometallic complexes with a metal in oxidation state zero and η^6^ coordination has been analyzed theoretically. The family presents four cases of complexes which yield promising results with the respect of thermodynamic stability, they are the complexes with fullerenes with symmetry C_2v_, D_3_, D_2_ and D_5d_ whereas the two remaining isomers coming from the more symmetrical fullerenes, *i.e.*, I_h_ and D_5h_, seems to be unstable species. All of them can have strong π bonds between the fullerene and the chromium atom, but the interaction is ruled by two factors, one is the narrow energy gap between HOMO and LUMO in all cases (which gives the character of conductive species to all isomers) that allow a back bond interaction that reinforce the bond and the pyramidalization angle that allow this back bond between the pz orbitals coming from the six membered ring of the fullerene that contributes to the bond (localized on the LUMO) and the dz^2^ orbital from the metal in the fragment CrC_6_H_6_ (localized in the HOMO). All species show interesting electronic features and if they are prepared will be useful for electronic devices application.
